# Health and Comfort

**Published:** 1875-10

**Authors:** 


### HEALTH AND COMFORT.

Health and comfort depend so largely in this northern latitude of ours upon the means employed in heating the places which we inhabit during the winter months, that we are justified in directing the attention of our readers to any device which appears to possess especial merit in this direction. There are stoves and heaters and fireplaces in abundance in the Fair, and certainly some very beautiful articles of this sort; but there is only one, the merits of which we have freely investigated and of which we can speak with the emphasis which attends conviction. We allude to Mr. A. M. Lesley's Gothic Furnace.

The regular readers of the *Journal* have long known our opinion of this excellent heating device. We instituted many enquiries among those who had this furnace in use, and published the testimony accumulated for the benefit of the large number of readers who asked us to investigate the subject in their behalf. The names of several leading citizens who used and praised the "Gothic," were published in our pages.

It is not with a view to repeat what we have before uttered, or particularly to enforce our earlier opinions as to the value of this furnace, that we now write. We merely wish to ask what sort of a structure a genuine, healthful, manageable heating appliance should be, and then to see whether the "Gothic" fulfills the indications.

A furnace for heating a house or church or any place which is inhabited, should have such close joints that no gas can by any possibility escape into the air-chamber, to be diffused with the heated air; it must have an ample fire-pot and large radiating surface; it must be easy to manage, and readily cleaned.

In our judgment the Gothic Furnace fulfills these conditions. The heavy gothic dome rests loosely in a deep groove, which is filled with fine sand. As one portion of the structure becomes hotter than another, unequal expansion occurs. Then the advantage of the sand-packing becomes apparent. The sand, though impervious to gas, readily yields to the enormous pressure of iron enlarged by heat, and is displaced at all points of expansion. The sand is still held within the groove, and follows the iron as it cools and supplies the places where the shrinkage occurs. At no point can gas escape into the hot-air chamber, and the quality of the castings would appear to preclude the possibility of any gas escaping through the dense and solid iron.

The fire-pot and radiating surfaces are large, which fact secures abundant heat without forcing the combustion of all the fuel possible to consume.

With an ample air-chamber copiously supplied with pure air from without, and with large flues and discharge-registers, a house, church, or store, may be kept at summer heat with a reasonable consumption of fuel, because the fire-pot does not need to be raised to a red heat in order to impart sufficient warmth. It is the tremendous forcing of the fires in our hot-air furnaces, by the ignorant attendants, which does the mischief. The vitality of the air is destroyed by contact with red-hot or white-hot iron, and it is quite inadequate to sustain life at its best. What is needed in a furnace, is the ability to supply a great deal of warm air, rather than a small amount of over-heated air; and this, moreover, is a grand feature in Mr. Lesley's furnace.

In ease of management, nothing better can be found. Since we are often compelled to trust to ignorant domestics, many of whom never saw a furnace, it is very important that the process of replenishing, and shaking down the ashes, and cleaning the fire-pot, and regulating the draft, shall be so simple that a child could not err in performing these various duties. It must be a stupid creature, indeed, who could prove so obtuse as not to be able to manage the Gothic Furnace, which is quite as simple and quite as easily cared for as a common stove, and requires no more skill and much less watching. It has an exceptionally convenient arrangement for clearing the grate of ashes, and for dumping its contents when desired.

These furnaces are arranged for wood as well as for coal, and those designed for wood admit huge sticks, four feet in length. These are splendid affairs for our northern timbered regions, and give great satisfaction wherever used.

Mr. Lesley has just completed a beautiful portable furnace on the plan of his large Gothic, which will, we think, find a great demand from the occupiers of houses of moderate size in city and country.

We had designed to speak of the Zero Refrigerator, another of Mr. Lesley's great inventions, but space fails us. Some of these Refrigerators may be seen at the Fair. Two of them are in the form of elegant sideboards, having the refrigerator department in the bottom, and fitted with convenient shelves precisely as sideboards usually are. We believe these elegant and useful articles of furniture will find a place in multitudes of homes, and we shall be glad if our allusions to them are effective in extending their sphere of usefulness. Mr. Alex. M. Lesley's factory s at 226 West 23rd Street New York, and circulars descriptive of his goods are mailed thence to those who can not call and [illegible] for themselves.

